# Evaluation of antimicrobial potential of free gallic acid and its polyvinyl-based nano-formulation

**DOI:** 10.1038/s41598-025-19519-0

**Published:** 2025-10-01

**Authors:** Habiba A. Ahmed, Asmaa Abdel-Fattah, Zeinab A. Salama, Abeer E. Abd El-Wahab

**Affiliations:** 1 Plant Biochemistry Department, National Research Centre, Dokki, Giza, 12622 Egypt; 2 Medical Biotechnology Department, Institute of Genetic Engineering and Biotechnology, City of Scientific Research and Technological Applications (SRTA-City), New Borg EL-Arab, Alexandria 21934 Egypt; 3 Pharmaceutical and Fermentation Industries Development Center, City of Scientific Research and Technological Applications (SRTA-City), New Borg EL-Arab, Alexandria 21934 Egypt

**Keywords:** Gallic acid nanoparticles, PVA, Antimicrobial activity, Biochemistry, Microbiology

## Abstract

This study explores the antimicrobial potential of gallic acid (GA) and gallic acid loaded with polyvinyl alcohol (PVA) nanocarriers as a good source of phenolic acids against various gram-positive bacteria including *Staphylococcus aureus* and *Streptococcus mutans* and gram-negative bacteria such as *Escherichia coli* and *Salmonella typhi*, as well as a fungal species (*Candida albicans*). Prior to evaluating their antimicrobial activity, the physical characterization of the GA-PVA-NPs was carried out using different techniques such as Fourier transform infrared spectroscopy (FTIR), thermogravimetric analysis (TGA), transmission electron microscopy (TEM), scanning electron microscopy (SEM), and dynamic light scattering (DLS). TEM imaging revealed nanoparticles with a uniform size and morphology, while DLS analysis confirmed the presence of nanoparticles with an average size of 128.1 ± 31.9 nm, indicating their potential for improved interaction with microbial cells. It is well established that nanoparticles around 100–150 nm can more easily penetrate bacterial cell walls, leading to membrane damage, oxidative stress through reactive oxygen species (ROS) generation, and disruption of vital cellular functions such as protein synthesis and DNA replication. These size-dependent mechanisms play a key role in enhancing the antimicrobial properties of nanoparticles. Following characterization, antimicrobial activity was assessed using the well diffusion technique and microplate reader assay. The microplate reader assay provided quantitative data, showing that gallic acid exhibited the highest inhibition against S. aureus (97.77%) and S. typhi (88.22%), while GA-PVA-NPs also demonstrated significant antimicrobial effects. The well diffusion technique confirmed these results, with inhibition zones ranging from 17.33 mm to 33.00 mm. The reduced effectiveness of the nanoparticles at lower concentrations may not necessarily undermine their potential, as the increased stability and controlled release properties of nanoparticles could provide long-term antimicrobial action that is beneficial for specific applications. These findings suggest that GA-PVA-NPs have significant potential as antimicrobial agents. Future studies will focus on investigating the stability and mode of action of these nanoparticles and exploring their application in food packaging for antimicrobial purposes, emphasizing their safety, effectiveness, and potential to extend shelf life.

**Supplementary Information:**

The online version contains supplementary material available at 10.1038/s41598-025-19519-0.

## Introduction

Gallic acid (GA), chemically known as 3,4,5-trihydroxybenzoic acid (C₇H₆O₅), is a naturally occurring bioactive compound widely regarded as safe for human use^1^. It is commonly found in traditional Chinese medicinal plants such as *Galla chinensis*, *Polygoni Multiflori Radix*,* Terminalia chebula Retz*,* and Rheum palmatum L.* In recent years, gallic acid has been recognized for its extensive bioactive properties, including antibacterial, antiviral, antifungal, anti-inflammatory, and antioxidant effects. These diverse properties have made it widely applicable across various fields, including food science, pharmaceuticals, and chemical industries^2,3^. Gallic acid is widely used in various countries and regions as a surface spray for aquatic products to prevent spoilage^4^. Beyond this application, it exhibits a range of biological activities, including antibacterial, anti-inflammatory, and antimutagenic properties^5^. Research by Liu et al. 2017^6^ revealed that gallic acid can inhibit biofilm formation by blocking the synthesis of polysaccharides essential for bacterial biofilms. This mechanism effectively curtails biofilm development in free-living *Shigella* and *S. aureus*. Additionally, another study demonstrated that nanomaterials synthesized using gallic acid and graphene exhibit strong pro-apoptotic effects on A-489 renal cell carcinoma cells^7^. This suggests potential therapeutic benefits for treating human renal epithelial cell carcinoma while sparing renal proximal epithelial cells. In this context, GA is anticipated to be a promising natural antibacterial agent. Despite this, recent research has shifted towards exploring its applications in polymer materials science and nanoscience. Examples include studies on acylated pectin with GA^8^, GA-loaded liposomes^9^, and GA conjugated with gold nanoparticles^10^. Nanotechnology has become an essential platform in the biomedical and pharmaceutical fields, offering significant improvements in the solubility, bioavailability, and targeted delivery of therapeutic agents. One of its promising applications is nano-encapsulation, which enhances the stability, controlled release, and biological effectiveness of various bioactive substances, particularly those derived from natural origins^11,12^. Polyvinyl alcohol (PVA) is a synthetic polymer that is biodegradable, biocompatible, and widely utilized in nanoparticle synthesis due to its strong film-forming ability, minimal toxicity, and favorable mechanical characteristics^13^. Encapsulating natural antimicrobial agents within PVA nanoparticles helps protect them from environmental degradation and improves their ability to interact with microbial membranes, thereby boosting their antimicrobial efficiency^14^. The increasing prevalence of antimicrobial resistance (AMR) poses a critical threat to global health, diminishing the efficacy of conventional antibiotics and prompting the need for alternative treatments^15^. In this context, plant-based antimicrobials have gained attention due to their abundance of bioactive constituents, such as polyphenols, flavonoids, and essential oils. Gallic acid, a naturally occurring phenolic compound, has demonstrated significant antimicrobial, antioxidant, and anti-inflammatory activities^16–18^. Nevertheless, its practical application is hindered by poor water solubility and chemical instability under physiological conditions. To address these limitations, encapsulation into PVA-based nanoparticles (GA-PVA NPs) offers a promising solution to improve its antimicrobial action and therapeutic value. The synthesis of GA-PVA -NPs in this study offers several advantages over conventional formulation methods. Using polyvinyl alcohol (PVA) as an encapsulating polymer enables the formation of stable, biocompatible, and biodegradable nanoparticles without the need for toxic solvents or harsh reaction conditions^19^. This green synthesis approach is cost-effective and scalable, making it suitable for industrial applications. Compared to other encapsulation techniques such as chemical cross-linking or emulsification using organic solvents this method minimizes potential degradation of gallic acid and preserves its bioactivity^20^. Additionally, the resulting nanoparticles provide controlled and sustained release of GA, enhance its solubility and stability, and improve its antimicrobial performance, particularly for applications in food preservation and biomedical materials. This study supports SDG 3 (Good Health and Well-being) by tackling antimicrobial resistance and contributes to SDG 12 (Responsible Consumption and Production) by encouraging the use of sustainable materials in the development of nanotechnology.

## Materials and methods

All chemicals used were of analytical grade. Gallic acid (≥ 98% purity, Cat. No. G7384) and polyvinyl alcohol (PVA, Mw ~ 85,000–124,000, 87–89% hydrolyzed, Cat. No. 363138) were purchased from Sigma-Aldrich (USA). Nutrient agar (Cat. No. CM0003) and potato dextrose agar (PDA, Cat. No. CM0139) were obtained from Oxoid Ltd. (UK). Deionized water was used in all experiments.

### Preparation of PVA stabilized GA-NPs

**Fig. 1:**
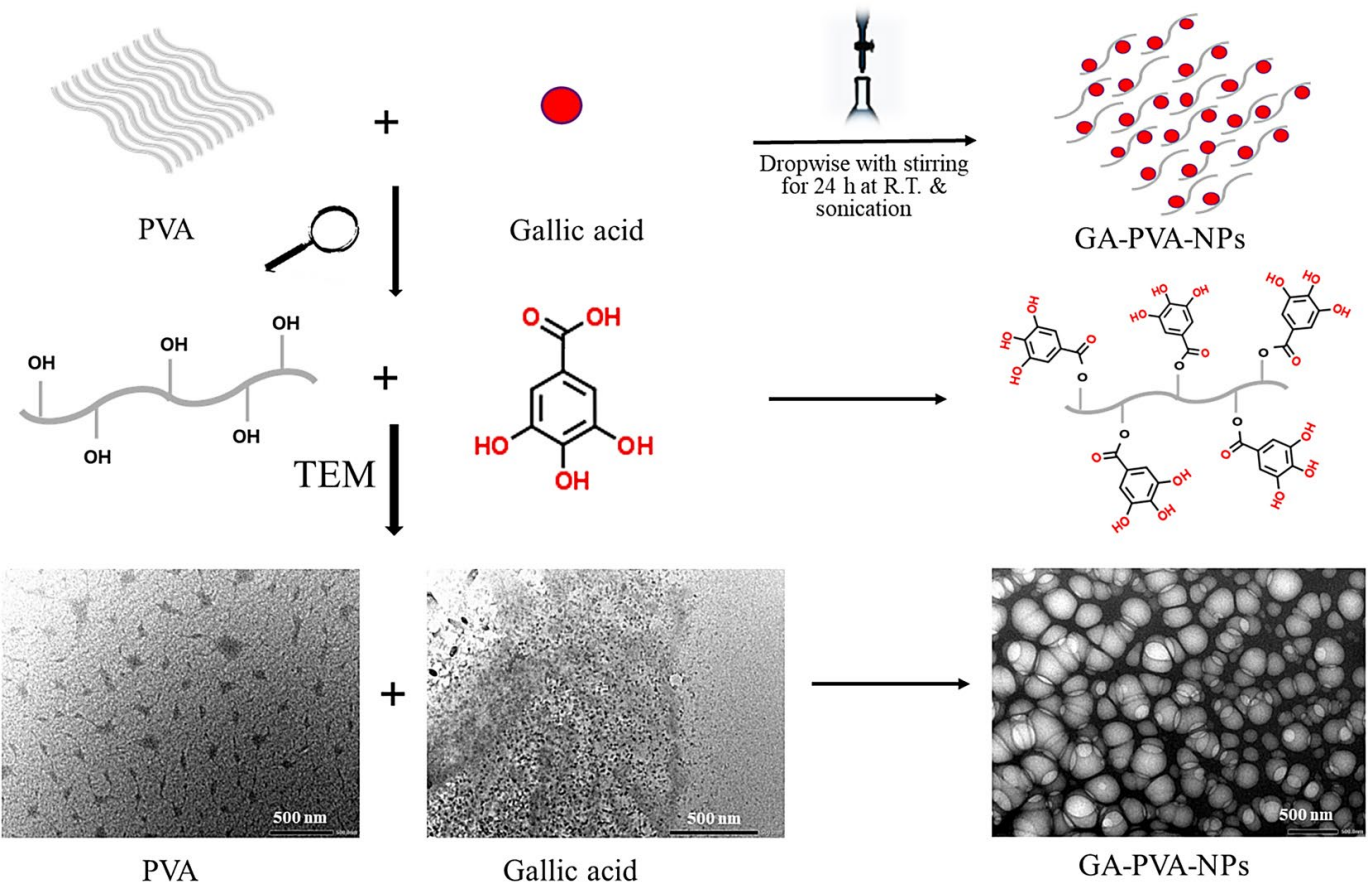
The scheme of the preparation of polyvinyl alcohol (PVA)-stabilized gallic acid nanoparticles (NPs), including TEM images of PVA, gallic acid, gallic acid-loaded PVA nanocarriers (GA- PVA-NPs).

Initially, 0.5 g of PVA was dissolved in 10 mL of distilled water under continuous magnetic stirring at 80 °C for 2 h. The solution was then maintained under stirring while cooling to room temperature (RT). For the incorporation of GA, a specified amount (0.1 g of active ingredient) was first dispersed in 10 mL of distilled water and stirred at RT for 1 h. This GA dispersion was then added to the polymeric PVA solution under magnetic stirring to ensure uniform mixing. To further enhance the homogeneity of GA in the aqueous solution, the mixture underwent sonication using an ultrasonic processor (DAIGGER ULTRASONIC Model GEX 750, USA) for 15 min. After sonication, the solution was stored at 4 °C until use^21^ (Fig. 1.)

### Characterization techniques

#### Fourier transform infrared spectroscopy (FTIR)

FTIR analysis was performed to identify functional groups and assess the extent of molecular interactions during nanoparticle formation. The spectra were recorded with a resolution of 4.0 cm⁻¹ across the wavelength range of 4000–400 cm⁻¹ using a Bruker Optics Tensor 27 spectrometer (Bruker Corporation, Billerica, MA, USA).

#### Transmission electron microscopy (TEM)

The structural characteristics and size distribution of the synthesized gallic acid nanocomposites were examined through TEM. Prior to imaging, the nanoparticle suspension was sonicated for 5 min to enhance dispersion.

#### Scanning electron microscopy (SEM)

SEM analysis was conducted to visualize the microstructural properties of the gallic acid nanocomposites. The lyophilized sample was mounted on an adhesive carbon stub, and imaging was performed using a JEOL 6340 tabletop SEM (Japan) at an accelerating voltage of 15 kV.

#### Thermogravimetric analysis (TGA)

Thermal stability assessments were carried out on both the control and gallic acid nanoparticles. The samples were heated from 0 to 1000 °C at a rate of 10 °C/min under a nitrogen atmosphere using a Themsys One Plus analyzer (SETARAM, France).

#### Zeta potential and particle size analysis

The nanoparticle size, polydispersity index (PDI), and surface charge were evaluated in aqueous suspension at room temperature using a Malvern Zeta Sizer (NanoZS, Malvern, UK).

### Antimicrobial activity

#### The well diffusion technique

The well diffusion technique was employed to evaluate the antimicrobial activity of gallic acid and gallic acid-PVA nanoparticles (GA-PVA NPs) at a concentration of 50 mg/mL against various microbial strains. Antimicrobial efficacy was assessed against a range of Gram-positive bacteria, including *S. aureus* (ATCC 13565) and *S. mutans* (ATCC 25175), as well as Gram-negative bacteria such as *E. coli* (ATCC 25922) and *S. typhi* (ATCC 14028). Additionally, antifungal activity was evaluated against the fungal species *C. albicans* (ATCC 10231). Bacterial susceptibility was determined by using nutrient agar medium, with plates incubated at 37 °C for 24 h. The inhibition zones were then measured in millimeters using a ruler. Similarly, antifungal activity was tested on potato dextrose agar (PDA), with inhibition zones recorded after 48 h of incubation at 25 °C^22^.

##### Microplate reader assay

Antibacterial activity was evaluated using a microplate reader assay to determine both the percentage of bacterial inhibition and the minimum inhibitory concentration (MIC), following the method of Bechert et al. (2000)^23^ with slight modifications. A 100 µL aliquot of pre-inoculated bacterial pathogens (10⁶ CFU/µL) in LB broth was transferred into each well of a 96-well plate. An equal volume (100 µL) of the test samples, gallic acid and gallic acid-PVA nanoparticles (GA-PVA NPs), was added to the respective wells in triplicate. The plates were then incubated under microaerophilic conditions at 37 °C for 24 h. After incubation, the absorbance of each well was measured at 620 nm using an automated ELISA microplate reader. The percentage of bacterial inhibition was calculated using the following equation: Inhibition (%)= (A​−A1​/A0-A1​​) × 100 where A is absorbance of the treatment group, A1 is the absorbance of the blank (media only, no cells or treatment) and A₀ is the absorbance of the control group (without treatment). The MIC was defined as the lowest concentration of the tested sample that resulted in no visible bacterial growth.

## Result and discussion

### Physical characterization

#### FTIR spectroscopy

**Fig. 2:**
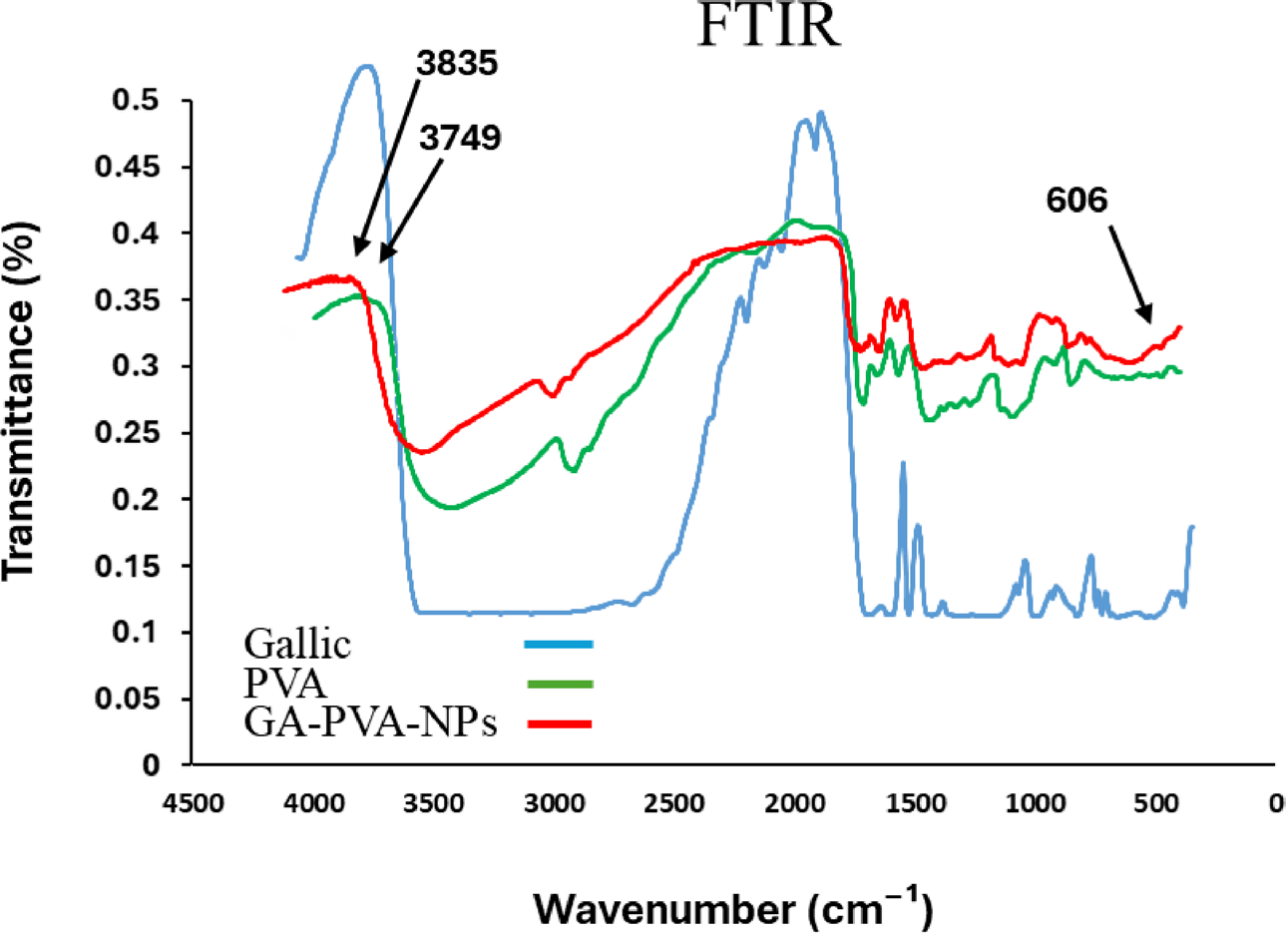
Fourier-transform infrared spectroscopy (FTIR) spectra of gallic acid (blue spectrum), PVA (green spectrum) and GA-PVA-NPs (red spectrum) The arrows point to new peaks.

Figure 2 illustrated the FTIR spectrum of gallic acid (GA) powder (blue spectrum), showing distinct absorption peaks. A broad peak at 3304 cm⁻¹ is attributed to O–H stretching vibrations. A sharp band at 1616 cm⁻¹ corresponds to the C = O stretching of the carboxylic acid group (–COOH). An absorption band at 866 cm⁻¹ is associated with O–H bending. The peak at 1537 cm⁻¹ is related to C = C stretching in the aromatic ring. Another peak at 1447 cm⁻¹ is attributed to C–C stretching and may also be associated with phenolic –OH bending^24^. The C–O stretching vibration is observed at 1200 cm, while additional bands in the region of 1300–1000 cm⁻¹ (1200, 1097, and 1028 cm⁻¹) are attributed to C–O bond stretching and O–H bending^25,26^. Minor signals corresponding to substituted benzene are detected at 959, 789, and 763 cm⁻¹^27^. For PVA (Fig. 2, green spectrum), the key absorption peaks include 3416 cm⁻¹, corresponding to O–H stretching, and 2915 cm⁻¹, associated with the asymmetric stretching of CH₂. A peak at 1654 cm⁻¹ indicates water absorption, while CH₂ bending appears at 1428 cm⁻¹. The band at 1329 cm⁻¹ is attributed to OH rocking combined with CH wagging. A shoulder peak at 1141 cm⁻¹ represents C–O stretching, signifying the crystalline structure of PVA. The peak at 1094 cm⁻¹, linked to C–O stretching and OH bending, reflects the amorphous regions of PVA. Additional peaks include CH₂ rocking at 919 cm⁻¹ and C–C stretching at 850 cm⁻¹^28,29^. The overlapping peaks observed in the FTIR spectrum of gallic acid-loaded PVA (Fig. 2, red spectrum), when compared to the individual spectrum of gallic acid and PVA, indicate an interaction between gallic acid and other nanomaterials. The appearance of new peaks at 3835, 3749, and 606 cm⁻¹ in the FTIR spectrum of gallic acid-loaded PVA nanoparticles indicates the formation of specific interactions between gallic acid and PVA matrix. The peaks observed at 3835 and 3749 cm⁻¹ are attributed to O–H stretching vibrations of free or weakly hydrogen-bonded hydroxyl groups^30^. These suggest that new hydrogen bonds may have formed, or existing ones may have been reorganized between the hydroxyl groups of gallic acid and the PVA polymer during the encapsulation process^31^. In contrast, the peak at 606 cm⁻¹ is likely related to bending vibrations of C–H bonds or deformation of the aromatic ring in gallic acid, indicating changes in its molecular structure resulting from interaction with the PVA matrix. These spectral changes confirm successful interaction and possible structural modification resulting from the nano-encapsulation process, in agreement with previous studies involving phenolic compounds and polymer-based carriers^30,32^.

### Transmission electron microscopy (TEM)

**Fig. 3:**
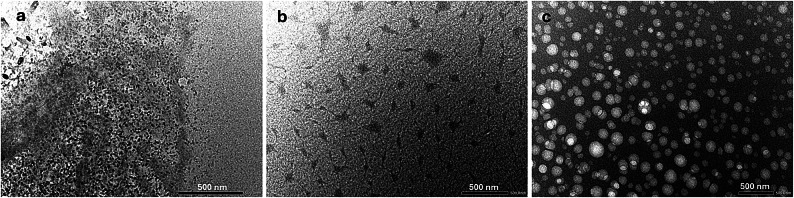
TEM images of (**a**) gallic acid, (**b**) polyvinyl alcohol (PVA), and (**c**) gallic acid-loaded PVA nanoparticles (GA-NPs), captured at the same magnification. Scale bars = 200 nm.

GA powder, as illustrated in Fig. 3a (appearance and chemical formula), is characterized by small, regularly shaped crystals that possess a smooth and uniform surface, indicating their high purity and consistent crystalline structure^33^. In contrast, Fig. 3b shows that the PVA polymer exhibited a network-like or chain-like structure, which can be attributed to its long molecular chains and the inherent flexibility and entanglement of its polymeric nature. This structural characteristic arises from the strong intermolecular hydrogen bonding between hydroxyl groups along the polymer backbone, allowing PVA to form interconnected networks or aggregated chains under certain conditions^34,35^. After preparing the GA-loaded PVA nanoparticles in water dispersion (Fig. 3c), the particles appeared as distinct entities, exhibiting a reduced diameter, and a spherical shape. The GA-loaded PVA nanoparticles were uniformly distributed, with their diameters falling within the range of 10 to 100 nm. This suggests a well-controlled synthesis process, crucial for achieving consistent nanoparticle size, which is important for enhancing their functionality in various applications^36^.

### Morphological properties

**Fig. 4:**
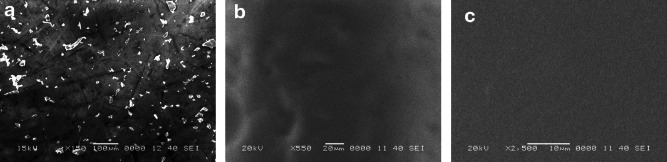
Micrographs of gallic acid (**a**), thin layer of PVA (**b**) and gallic acid -loaded PVA nanoparticles (**c**) at different magnifications.

Figure 4 illustrates the morphology of gallic acid, fractured sections of PVA, and GA-loaded PVA formulations. It also highlights the crystallinity of gallic acid (Fig. 4a), where the crystals show a well-ordered structure with sharp, angular shapes. The surface of the gallic acid particles appear smooth, with some agglomeration typical of crystalline forms. The PVA film (Fig. 4b) exhibits a uniform, smooth, and consistent fractured surface, as noted in previous studies^37,38^. This feature underscores the excellent processability and film-forming ability of PVA during the solvent casting process. In Fig. 4c, the incorporation of GA influences the uniformity and consistency of PVA films. When GA is blended into the polymer matrix via solvent casting, it disperses evenly, likely due to its strong affinity for water and solubility in aqueous solutions^39^.

### Particle size distribution

Table 1 showed that gallic acid and PVA individually exhibited particle sizes of 256 nm and 518 nm, respectively, whereas the nanoparticles formed by incorporating gallic acid into PVA showed a significantly smaller size of 128 nm. The polydispersity index (PDI) serves as a measure of particle size uniformity, indicating how much the sizes of individual particles deviate from the average. A lower PDI value signifies a narrower size distribution, with a PDI of 0.5 or below considered ideal for uniformity^40^. In this study, the PDI for gallic acid and PVA alone was 1.0 and 0.5, respectively, while the PDI for the gallic acid-loaded PVA nanoparticles was 0.5, confirming a well-distributed particle size. This consistency highlights the reliability and reproducibility of the method in producing nanoparticles with a uniform size and distribution. The zeta potential values of gallic acid and PVA nanoparticles individually were shown in Table 1 (-9.76 mV and − 14.9 mV, respectively), while the zeta potential of gallic acid-loaded PVA nanoparticles was significantly reduced to − 0.71 mV. This decrease in negative surface charge could be attributed to gallic acid’s multiple hydroxyl groups, which ionize and release protons depending on the pH. These negatively charged groups likely interact with the negatively charged PVA surface, leading to partial neutralization or masking of surface charges and, consequently, a lower overall zeta potential^38,41,42^. Particle size distribution and zeta potential analyses are presented in Supplementary Figures S7–S12. Specifically, particle size distribution is shown in Figures S7–S9 for PVA, gallic acid, and nano-gallic acid, respectively, while zeta potential measurements are provided in Figures S10–S12 for gallic acid, nano-gallic acid, and PVA, respectively.

**Table 1:** Particle size, polydispersity index and zetapotential of gallic acid, PVA, gallic acid-loaded PVA.

	Gallic acid	PVA	GA-PVA-NPs
Z-Average (d.nm)	256.6	518.6	128.1 ± 31.9
Pdi	1.00	0.577	0.500
Zeta potential	− 9.76	− 14.9	-0.71

### The thermal stability (TGA)

**Fig. 5:**
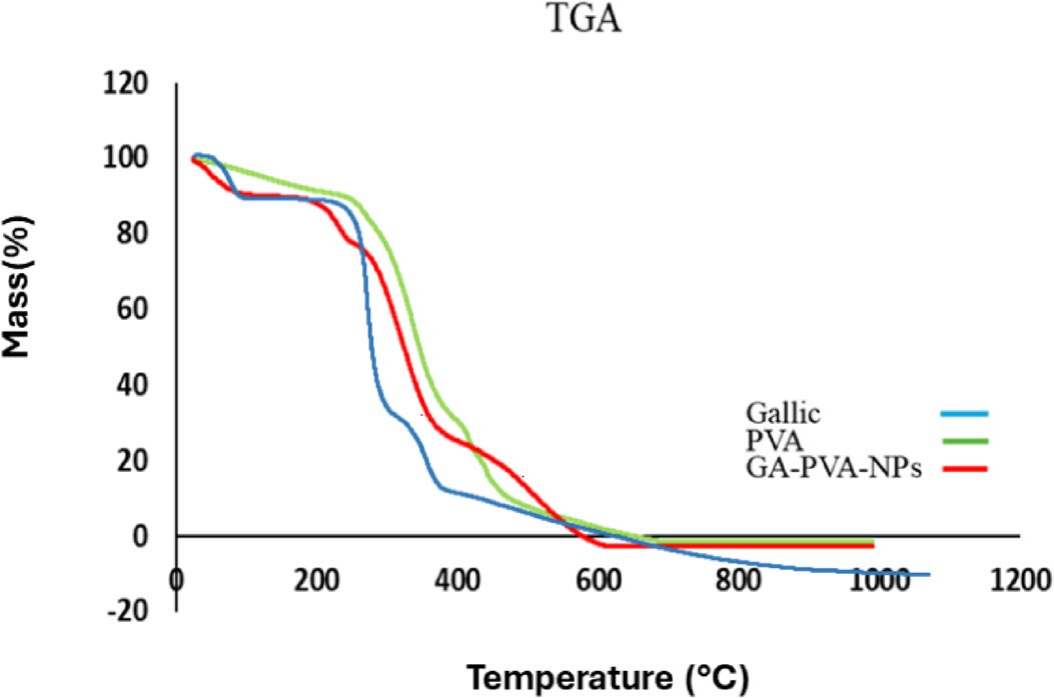
Thermogravimetric analysis (TGA) spectra of gallic acid (blue spectrum), PVA (green spectrum) and GA-PVA-NPs (red spectrum).

Gallic acid (GA), known for its hygroscopic nature, displayed an initial mass loss of 0.7% around 110 °C (Fig. 5, blue spectrum), attributed to the release of water molecules from its hydrate crystal structure^43,44^. Beyond this point, GA remained thermally stable until 217 °C, marking the onset of degradation^45^. At 297 °C, a significant mass loss of 54% occurred, corresponding to a sharp exothermic reaction. Additional mass losses were observed at elevated temperatures, reducing the mass by 21% at 372 °C and by 7% at 500 °C. For PVA (Fig. 5, green spectrum), the first degradation event, observed as a low-intensity peak between 100 and 239 °C, resulted in a 10% mass loss, which is attributed to the loss of water. A more prominent degradation peak was observed at 384 °C, where a 56% mass loss occurred, signifying the breakdown of the polymer’s main chain^46^. As the temperature increased, additional mass losses were noted, with a reduction of 11% at 424 °C and a further 13% at 481 °C. For the nanoparticles of GA-loaded PVA (Fig. 5, red spectrum), the initial mass loss of 12% occurred at 239 °C, followed by a significant mass loss of 51% at 399 °C. A further reduction of 18% in mass was observed at 531 °C during the final stage of degradation. When gallic acid is loaded into PVA nanoparticles, the gallic acid molecules are physically encapsulated within the polymer matrix. This encapsulation prevents direct exposure to environmental factors such as temperature, moisture, and oxygen, which can degrade the compound. This protective barrier increases the overall stability of gallic acid^47^.

### Antimicrobial activity

#### Well diffusion technique

**Fig. 6:**
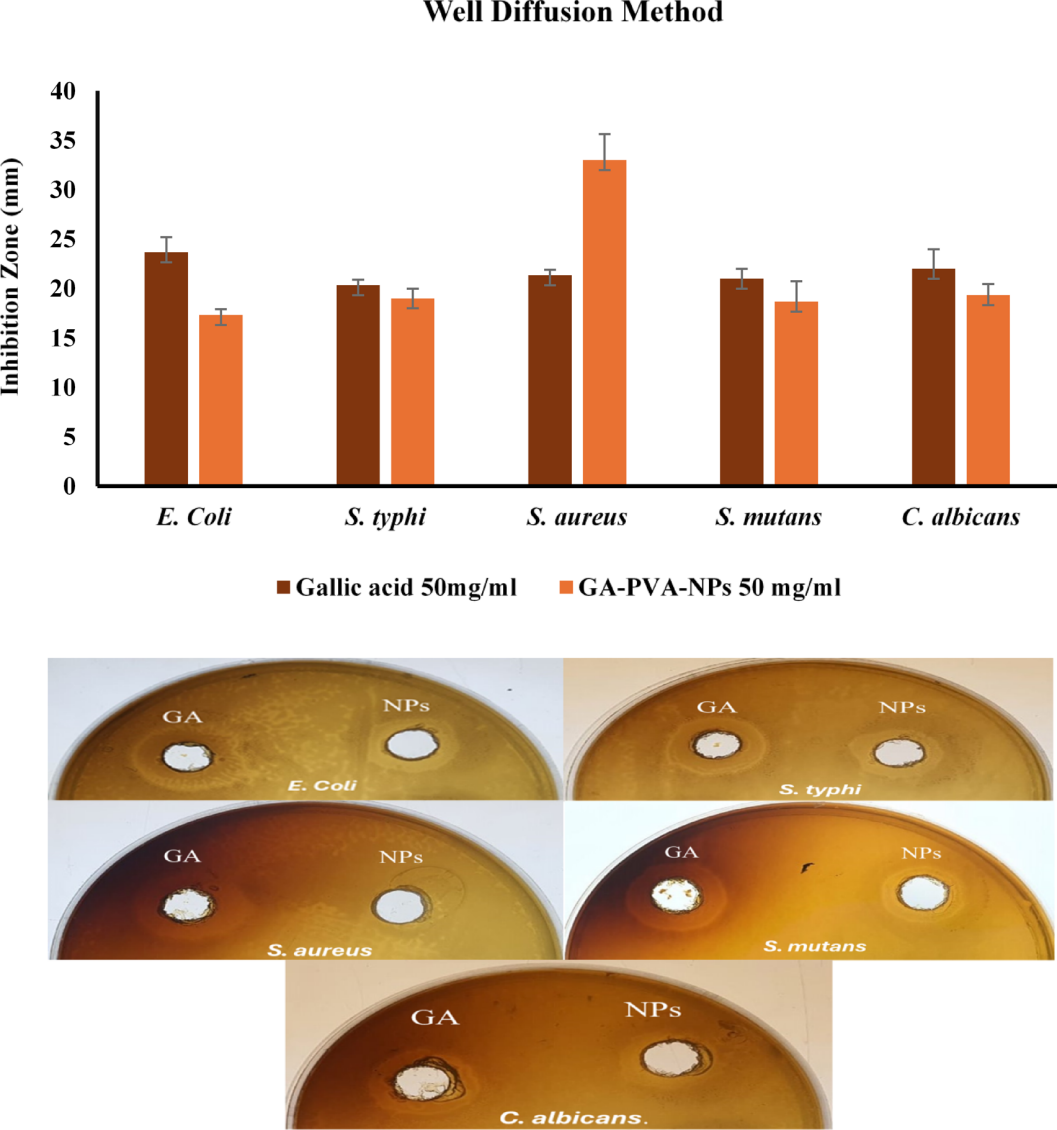
Inhibition zones of gallic acid and GA-PVA-NPs (50 mg/mL) against tested microbes using a well diffusion method.

Figure 6 illustrated the antimicrobial effects of gallic acid and its nano-formulated (GA-PVA-NPs) against several microbial strains. Both formulations exhibited notable inhibitory activity against all tested strains. However, no statistically significant differences were detected in their effects against *C. albicans* (22.00 mm for gallic acid vs. 19.33 mm for GA-PVA-NPs) and *S. mutans* (21.00 mm vs. 18.67 mm), suggesting similar efficacy in these cases. In contrast, GA-PVA-NPs showed significantly greater inhibition of *S. aureus* (33.00 mm), compared to gallic acid alone (21.33 mm), highlighting the benefit of nano-encapsulation in enhancing antibacterial potency, particularly against Gram-positive bacteria. On the other hand, gallic acid was more effective than GA-PVA-NPs against the Gram-negative strains *E. coli* (23.67 mm vs. 17.33 mm) and *S. typhi* (20.33 mm vs. 19.00 mm), likely due to its smaller molecular size and greater ability to diffuse through the protective outer membranes of these bacteria^48^. This strong antimicrobial effect can be attributed to gallic acid’s ability to disrupt bacterial cell membranes and interfere with essential bacterial metabolic pathways, making it a potent compound for inhibiting bacterial growth^49^. These observations are consistent with earlier reports that emphasize the advantages of nanoparticle systems in improving antimicrobial activity through enhanced cellular interaction^50^, better membrane penetration, and sustained release especially against Gram-positive pathogens. From a food packaging perspective, the superior performance of GA-PVA-NPs against *S. aureus* suggests promising potential for their incorporation into active packaging films to prevent contamination. Although slightly less effective against certain strains in short-term assays, the controlled-release nature of GA-PVA-NPs may offer longer-lasting antimicrobial protection, a highly desirable feature in food preservation applications^51–53^.

The antibacterial mechanism or free gallic acid or GA-PVA nanoparticles (GA-NPs), like other nanomaterial-based therapeutics, involves multiple pathways that directly disrupt bacterial viability. One key mechanism is the interaction between the nanoparticle surface and the negatively charged bacterial membrane, leading to membrane depolarization, increased permeability, and eventual structural collapse^54^. Additionally, GA-NPs can induce the generation of reactive oxygen species (ROS), which cause oxidative damage to vital cellular components such as lipids, proteins, and nucleic acids, disrupting normal metabolic functions. Some nanomaterials may also physically damage bacterial cell walls through sharp-edge effects or surface tension-based rupture. Moreover, in polymer-based systems, the sustained release of active compounds like gallic acid enables prolonged exposure to antimicrobial agents, enhancing the inhibitory effect^55,56^. These mechanisms can act synergistically, contributing to the broad-spectrum antibacterial activity of nanomaterials. Importantly, the multi-target nature of these actions reduces the likelihood of resistance development, making GA-PVA-NPs a promising candidate not only for food packaging applications but also for future biomedical uses. On the other hand, the antibacterial mechanisms of GA-PVA nanoparticles appear to vary between Gram-positive and Gram-negative bacteria due to fundamental differences in their cell wall structures. Gram-positive bacteria, such as *S. aureus* and *S. mutans*, have a thick peptidoglycan layer that is directly exposed to the external environment, making them more susceptible to surface interaction and physical disruption by nanoparticles. GA-PVA-NPs may readily bind to and destabilize this layer, leading to leakage of cytoplasmic contents and cell death^57,58^. In contrast, Gram-negative bacteria like *E. coli* and *S. typhi* possess an additional outer membrane composed of lipopolysaccharides, which acts as a protective barrier and can limit the penetration of nanoparticles^59^. As a result, the antimicrobial activity of GA-PVA-NPs against Gram-negative strains may be less efficient due to restricted access to the inner cell membrane and reduced interaction with intracellular targets. These structural differences help explain the higher sensitivity observed in Gram-positive bacteria and underscore the importance of cell wall composition in determining nanoparticle efficacy^60,61^.

#### Microplate reader assay

**Fig. 7:**
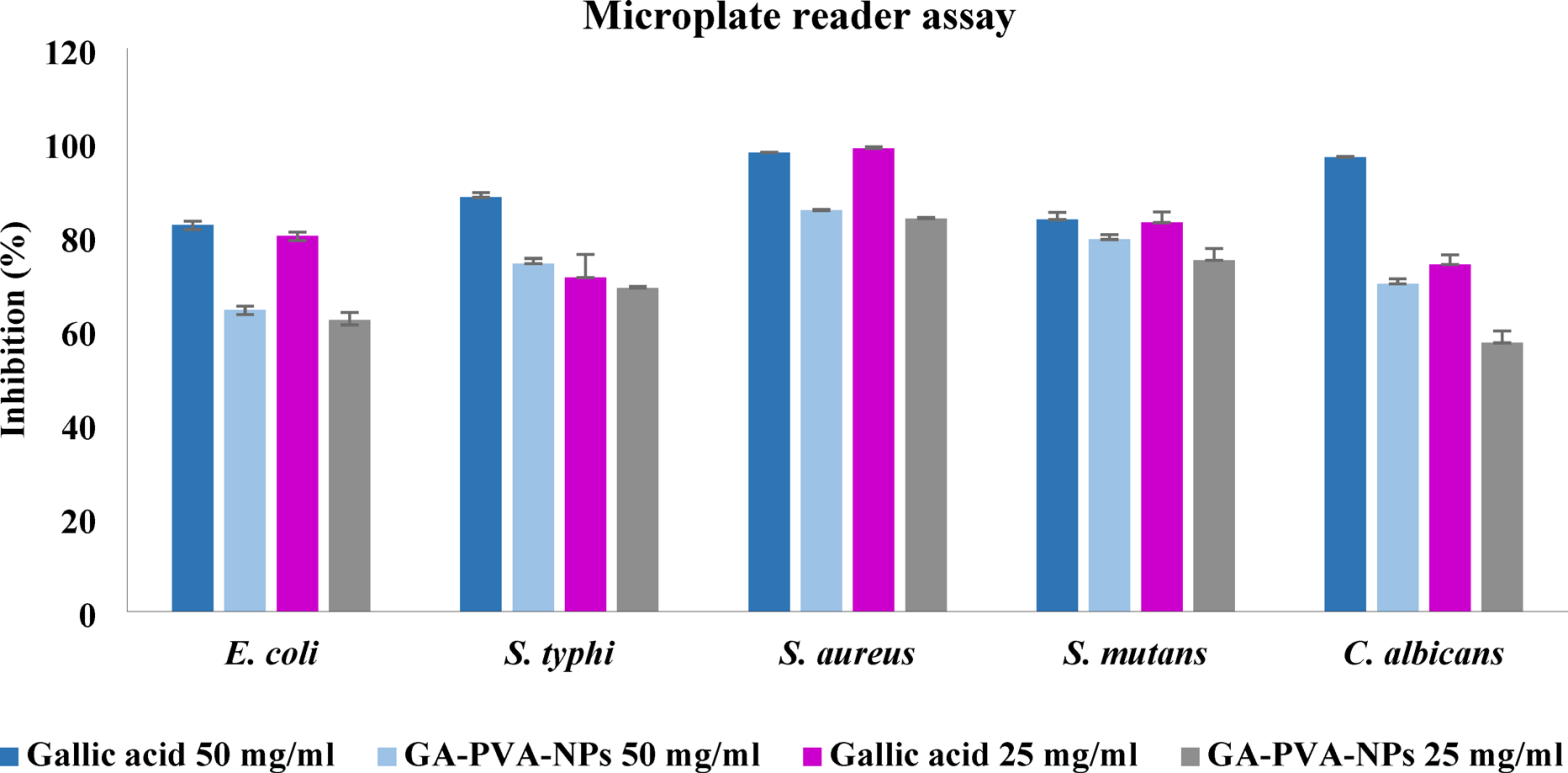
Antimicrobial activity of gallic acid and GA-PVA-NPs, using the microplate reader assay, tested at varying concentrations (50 and 25 mg/ml).

The antimicrobial activity of gallic acid and its nanoparticles (NPs) was evaluated using the well diffusion method to measure inhibition zones. Among the tested concentrations, 50 mg/mL was the lowest that produced a visible inhibition zone, making it suitable for comparison with results obtained from the microplate reader, which provided corresponding percent inhibition values. In contrast, a lower concentration (25 mg/mL) did not produce any visible zone in the well diffusion assay but showed a clear inhibitory effect when assessed using the microplate reader. These findings support the decision to continue using serial dilutions to determine the minimum inhibitory concentration (MIC) via the microplate reader assay, which offers greater sensitivity for detecting antimicrobial activity at lower concentrations. This may be attributed to the fact that the microplate reader assay provides direct contact between the antimicrobial agent and microorganisms in liquid media, avoiding the diffusion limitations of agar-based methods that can underestimate activity^62^. Figure 7 shows the antimicrobial activity assessed by the microplate reader assay at two concentrations, 25 and 50 mg/mL. The results indicated no significant difference between the two concentrations against all tested strains. However, when comparing gallic acid and its nanoparticles at the same concentration, noticeable differences in antimicrobial activity were observed. The microplate reader assay revealed that free gallic acid exhibited significantly higher inhibition percentages against all tested strains. The microplate reader assay demonstrated that free gallic acid exhibited superior antimicrobial efficacy compared to its nanoparticle formulation (GA-PVA-NPs) against all tested microbial strains. Among Gram-negative bacteria, *E. coli* showed an inhibition rate of 82.23% with gallic acid, whereas GA-PVA-NPs achieved 64.33%. Similarly, *S. typhi* exhibited 88.22% inhibition with gallic acid and 74.12% with its nano-formulation. In the case of Gram-positive bacteria, *S. aureus* displayed the most significant response, with 97.77% inhibition by gallic acid compared to 85.45% by GA-PVA-NPs. *S. mutans* followed a similar pattern, with 84.36% and 79.26% inhibition for gallic acid and GA-PVA-NPs, respectively. Furthermore, *C. albicans* demonstrated high susceptibility to gallic acid (96.81%), while GA-PVA-NPs resulted in a lower inhibition rate of 69.82%. These findings indicate that free gallic acid possesses greater antimicrobial potential in broth-based assays, likely due to its higher immediate bioavailability and direct interaction with microbial cells. In contrast, the encapsulated form may exhibit delayed or incomplete release of the active compound under these conditions, thereby reducing its effectiveness. This discrepancy contrasts with results obtained from the well diffusion method, where nanoparticle formulations may perform more favorably due to enhanced diffusion and controlled release within the agar matrix^62,63^. The encapsulation of gallic acid in PVA nanoparticles can improve its stability, protect it from environmental degradation, and allow for sustained release over time an important feature for maintaining antimicrobial effectiveness in food contact materials^64,65^. Therefore, GA-PVA-NPs hold potential as a functional component in active food packaging systems aimed at extending shelf life and inhibiting microbial contamination.

#### Minimum inhibitory concentration

**Fig. 8:**
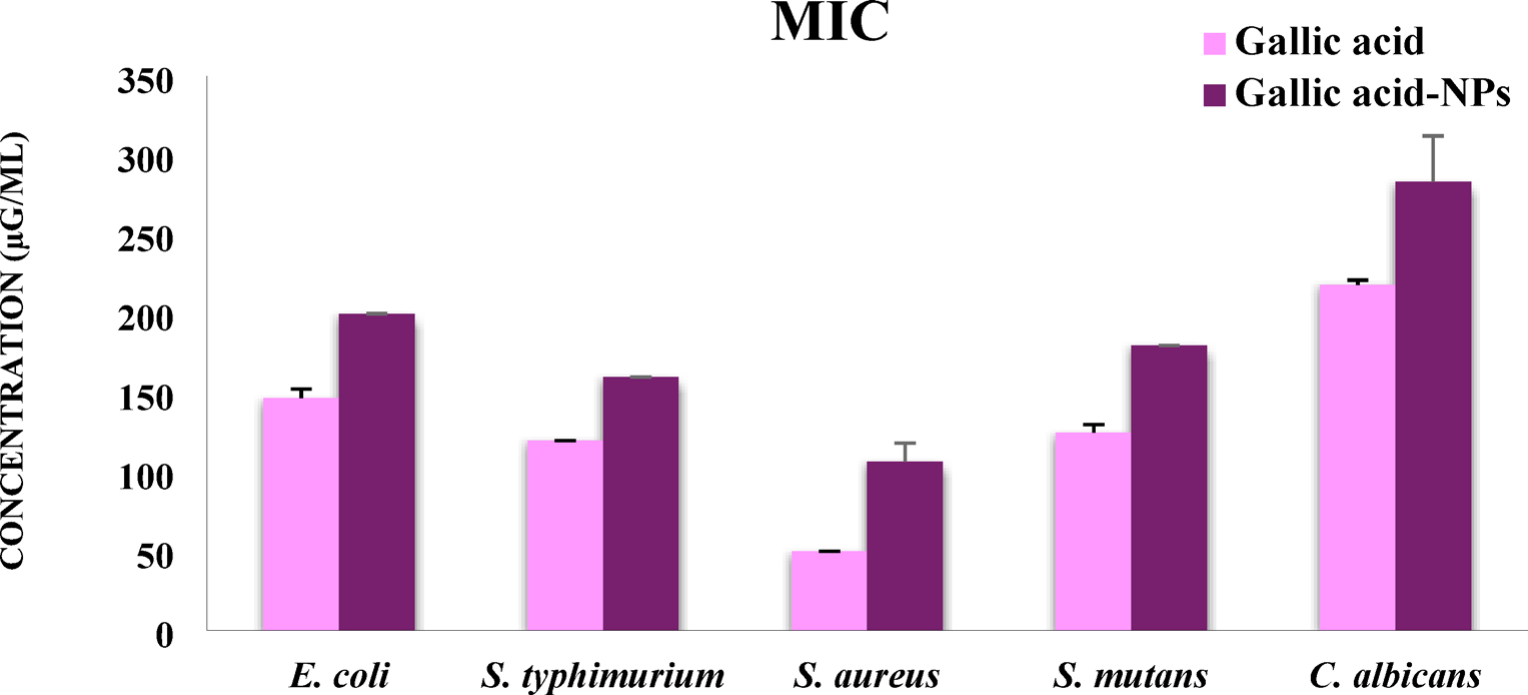
Minimum inhibitory concentrations (MIC) of gallic acid and gallic acid-loaded PVA nanoparticles (GA-PVA-NPs) against selected microbial strains.

Minimum inhibitory concentration (MIC) values are presented in Fig. 8. The MICs of gallic acid against the tested strains were 147 µg/mL for *E. coli*, 120 µg/mL for *S. typhi*, 50 µg/mL for *S. aureus*, 125 µg/mL for *S. mutans*, and 218 µg/mL for *C. albicans*. In comparison, the MICs of the nano-formulated gallic acid (GA-PVA-NPs) were higher for all strains: 200 µg/mL for *E. coli*, 160 µg/mL for *S. typhi*, 107 µg/mL for *S. aureus*, 180 µg/mL for *S. mutans*, and 283 µg/mL for *C. albicans*. These results indicate that free gallic acid has a stronger inhibitory effect than its nano- formulation under the tested conditions. The MIC results revealed that free gallic acid exhibited lower MIC values compared to its nanoform (GA-PVA-NPs) across all tested strains, indicating stronger antimicrobial potency. This difference may be attributed to the immediate bioavailability of free gallic acid in the broth medium, whereas the nanoform may release the active compound more slowly. Although nanoparticles offer advantages like improved stability and sustained release, these findings suggest that, under the tested conditions, free gallic acid is more effective for rapid microbial inhibition^66,67^. In the current study, structural changes in bacterial cells were not examined. However, since the antimicrobial activity of GA-PVA nanoparticles (GA-NPs) is concentration-dependent, it is reasonable to assume that higher concentrations may lead to more pronounced damage to bacterial cell walls and membranes. In future studies, we plan to investigate these structural changes using imaging techniques such as scanning or transmission electron microscopy (SEM/TEM) to better understand the mode of action of GA-NPs at the cellular level. This will help clarify how different concentrations of GA-NPs influence bacterial morphology and contribute to their antimicrobial effects.

## Conclusion

This study highlights the potent antimicrobial activity of gallic acid and its nano-formulated counterpart (GA-PVA-NPs) against a broad spectrum of microbial strains. Both formulations demonstrated significant inhibition across Gram-positive, Gram-negative bacteria, and fungi; however, their efficacy varied depending on the assay method and microbial target. Free gallic acid consistently outperformed its nanoparticle form in broth-based (microplate reader) assays, likely due to its immediate solubility and faster interaction with microbial cells. Conversely, GA-PVA-NPs exhibited superior performance in agar-based diffusion assays, particularly against *S. aureus*, suggesting enhanced diffusion and sustained release capabilities. Minimum inhibitory concentration (MIC) values further confirmed that free gallic acid exhibited stronger antimicrobial potency, especially at lower concentrations. Nevertheless, the nanoform maintained appreciable activity, supporting its application where long-term, controlled antimicrobial action is desirable. Given its improved stability, gradual release profile, and proven antimicrobial effects especially against Gram-positive pathogens, GA-PVA-NPs demonstrate promising potential for use in active food packaging systems to enhance food safety and shelf life. In future studies, we aim to apply GA-PVA-NPs in food packaging films and evaluate their antimicrobial performance under real storage conditions. Additionally, we will investigate the physicochemical stability and release behavior of the nanoparticles to optimize their functional properties for industrial applications. Given their biocompatibility and antimicrobial efficacy, GA-PVA-NPs may also hold promise for biomedical applications such as wound dressings or antimicrobial coatings.

## Supplementary Information

Below is the link to the electronic supplementary material.

Supplementary Material 1

Supplementary Material 2

## Data Availability

Data Availability Statement: Data is provided within the manuscript and supplementary information files. also, the raw data supporting the findings of this study are provided in the supplementary file in the submission system.
