# Supplementary material for: Evaluation of antimicrobial potential of free gallic acid and its polyvinyl-based nano-formulation
Source: Sci Rep. 2025 Oct 1;15:34281. doi: 10.1038/s41598-025-19519-0 (PMC12488905; doi:10.1038/s41598-025-19519-0)
Supplement: Supplementary file 1 — Supplementary Material 1 [file 41598_2025_19519_MOESM1_ESM.pdf]

Sample: 1 DR HABIBA
Size: 1.6954 mg

Comment: 10C/MIN

SDT

File: F:\SARAH\2024\4-11-2024\1 DR HABIBA.UA
Operator: SARAH
Run Date: 04-Nov-2024 12:56

Supplementary Figure S1

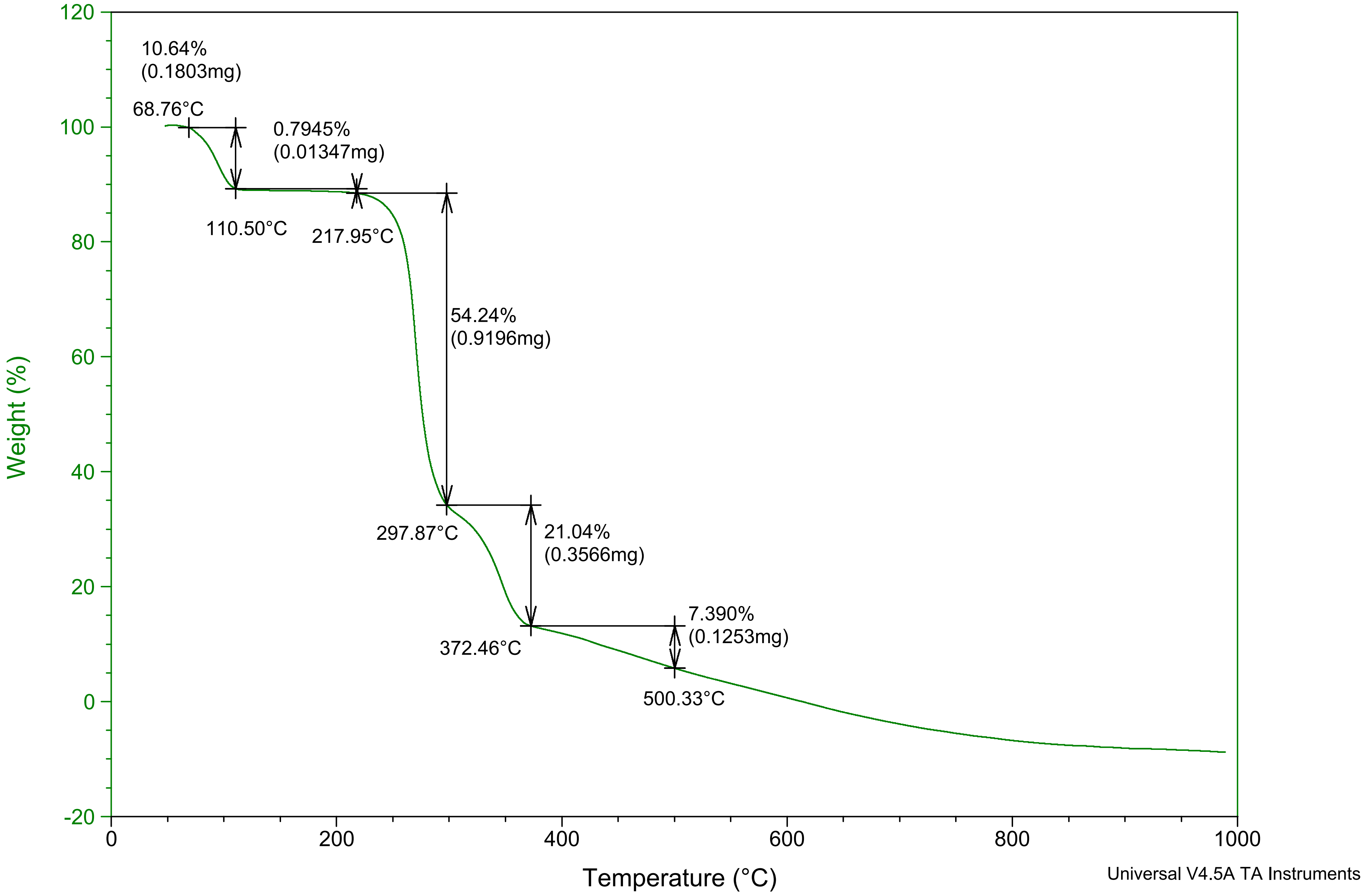

Sample: 3 DR.HABIBA
Size: 1.9098 mg

Comment: 10C/MIN

SDT

File: F:\SARAH\2024\13-11-2024\3 DR.HABIBA.UA
Operator: SARAH
Run Date: 13-Nov-2024 10:38

Supplementary Figure S2

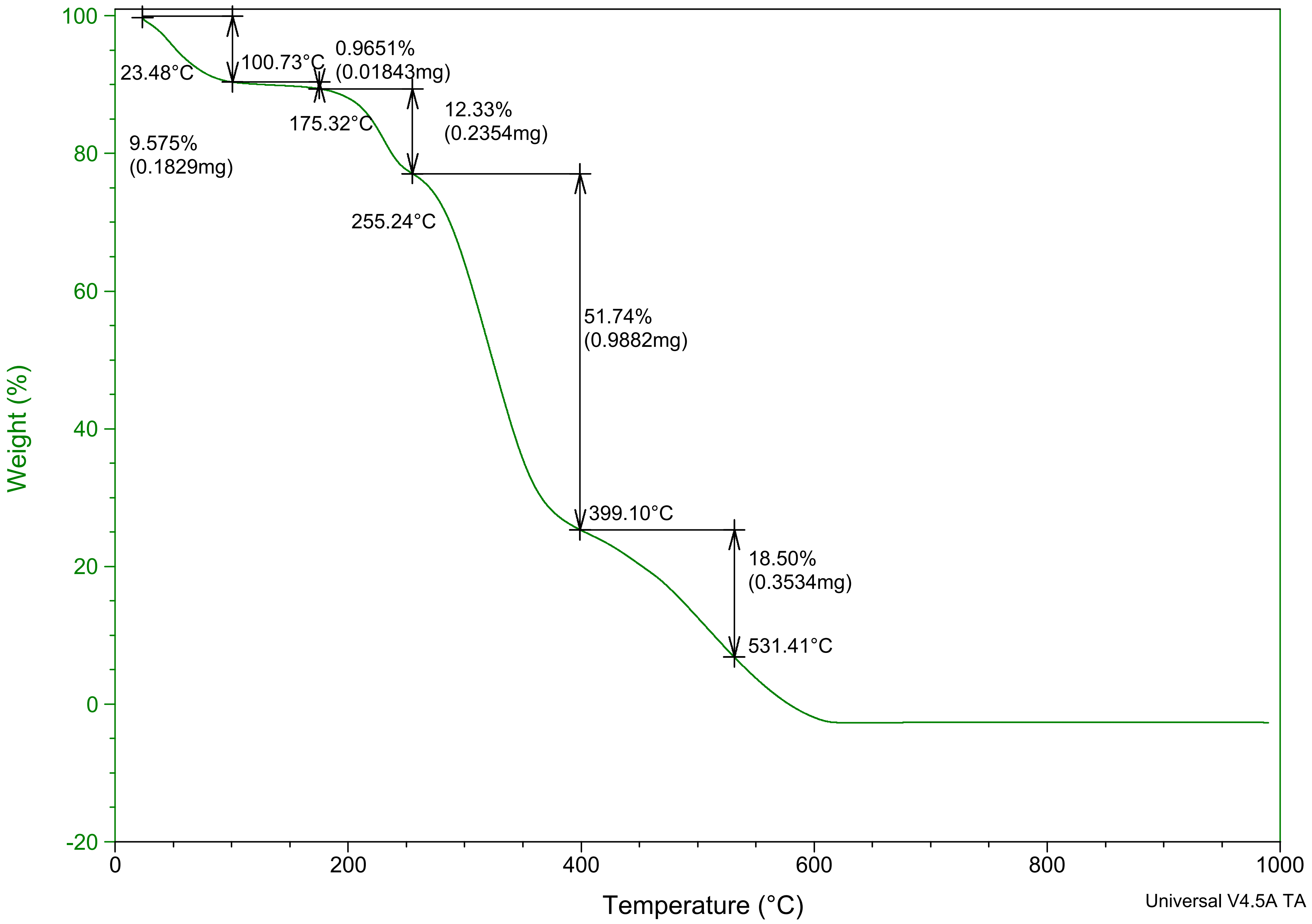

Universal V4.5A TA Instruments

Supplementary Figure S3

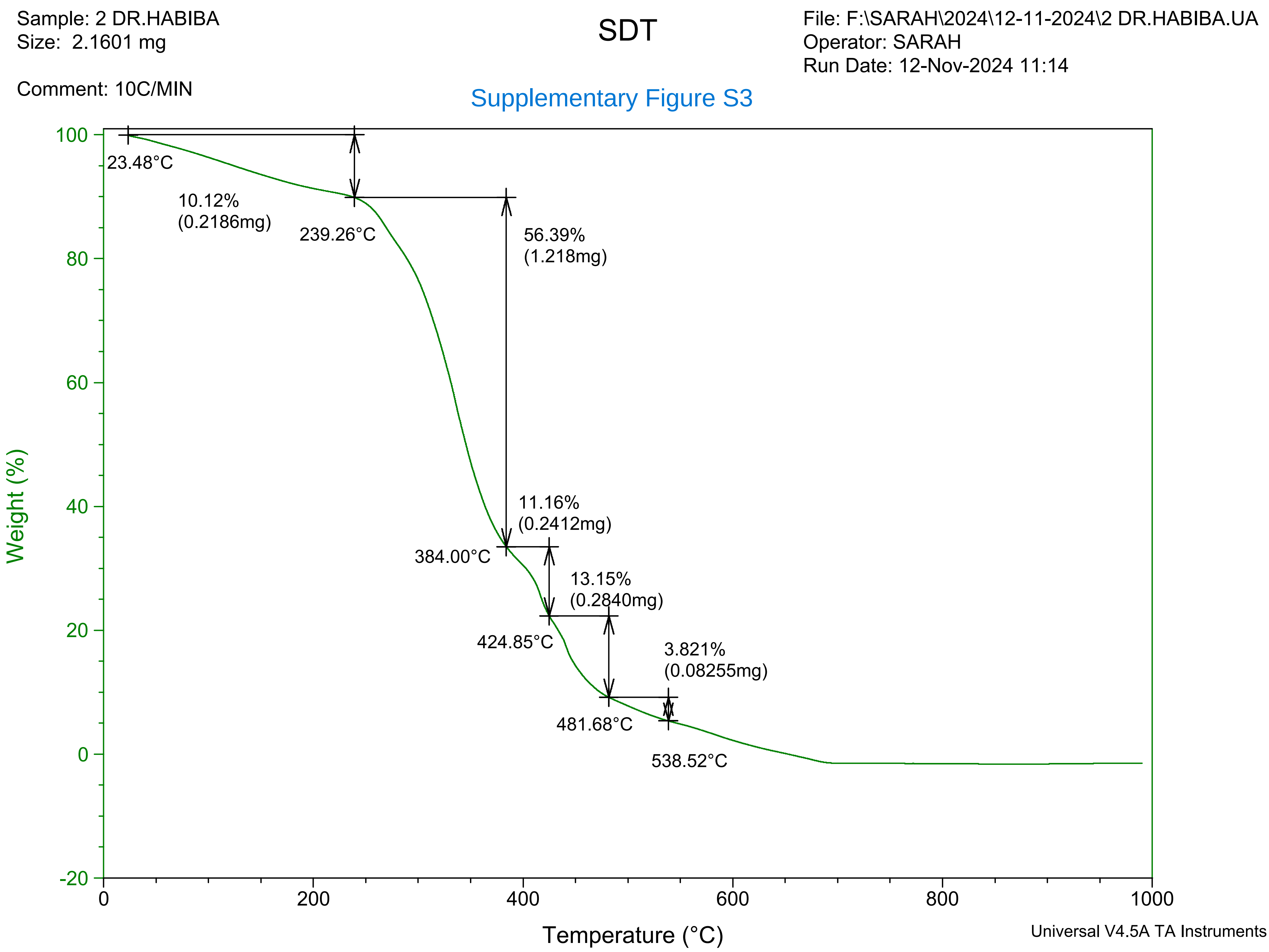

Supplementary Figure S4

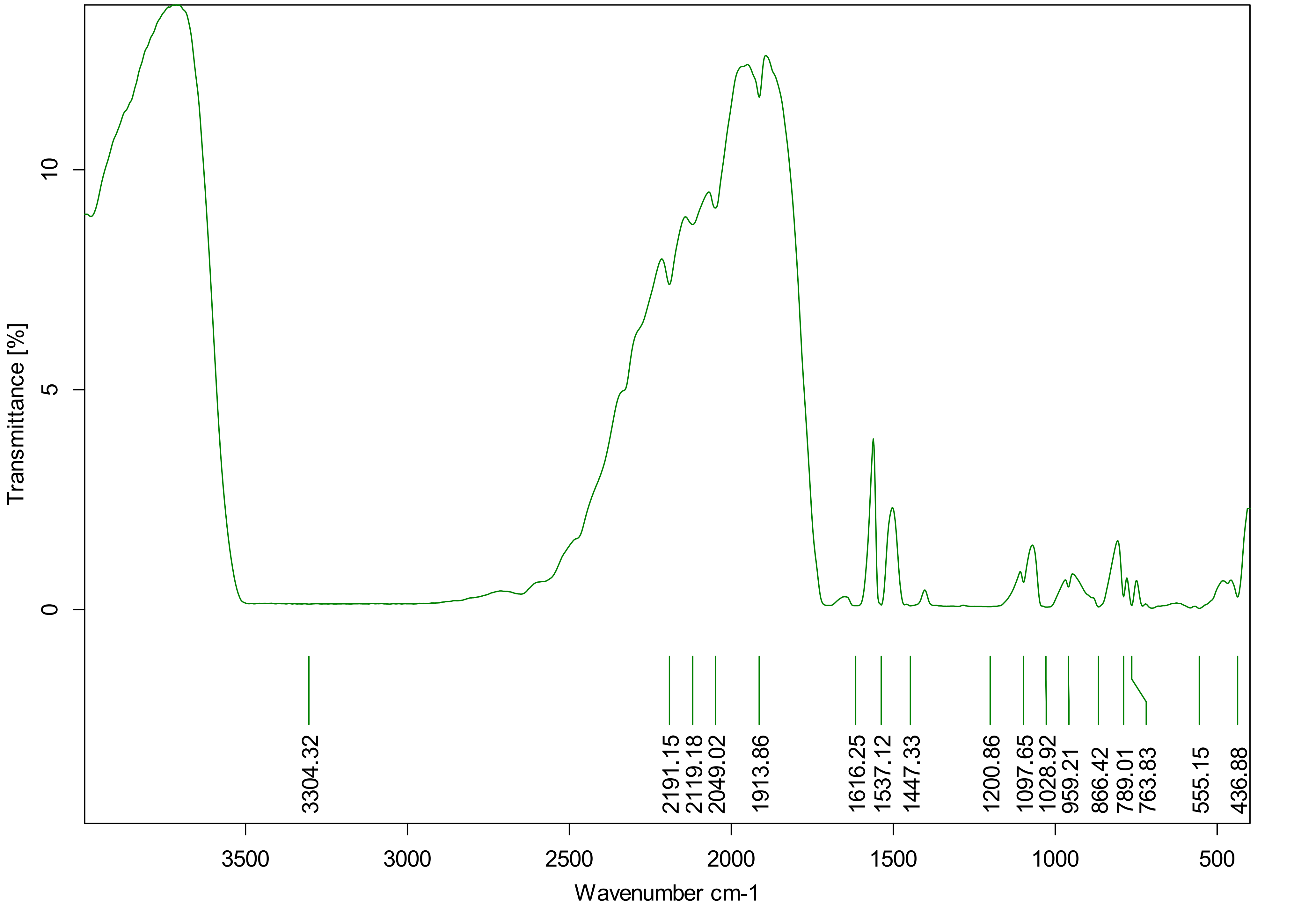

Supplementary Figure S5

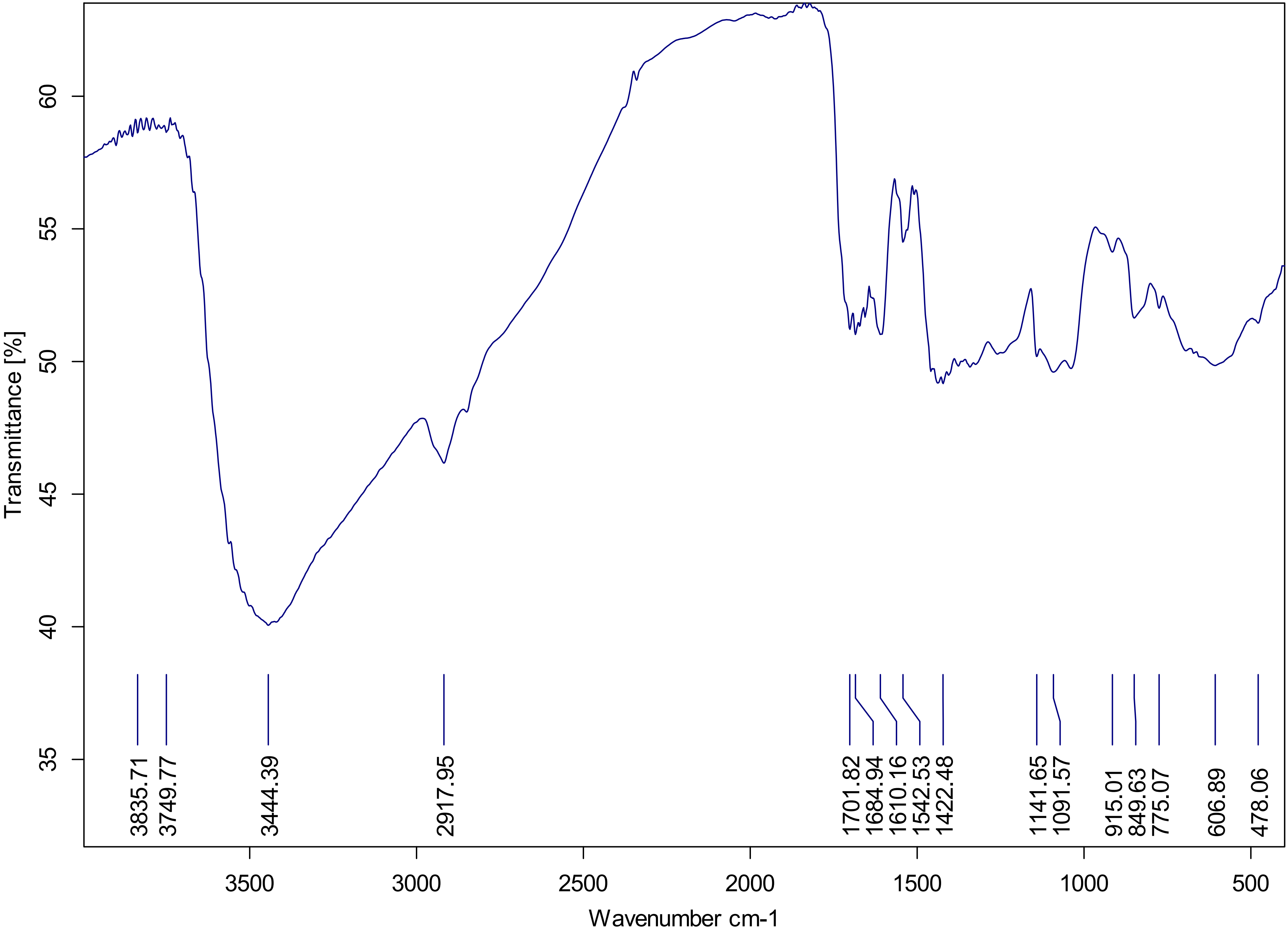

Supplementary Figure S6

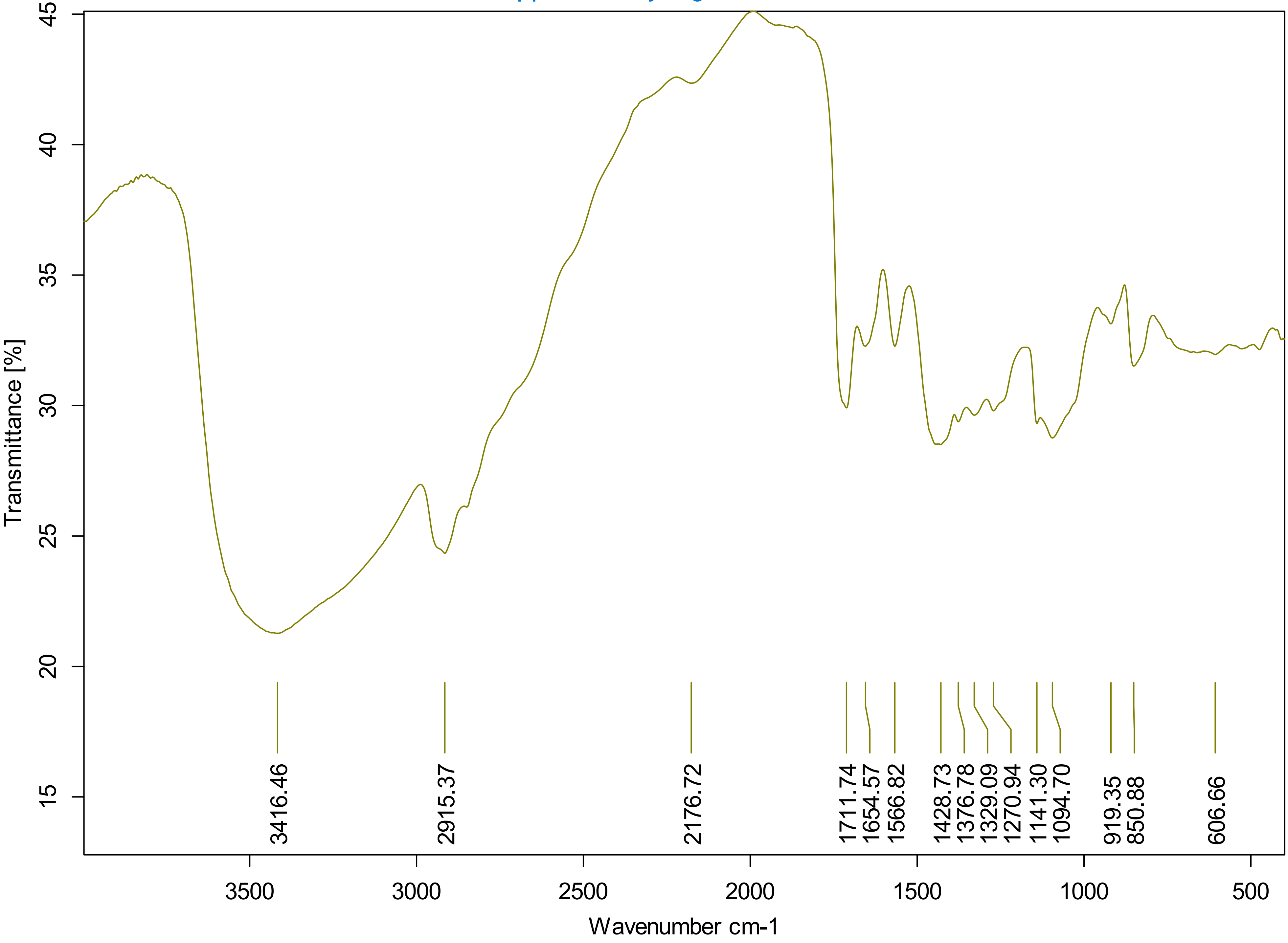

Size Distribution Report by Intensity

v2.2

Supplementary Figure S7

Malvern

Sample Details

Sample Name: 1 size 1

SOP Name: Size Water 0.3

General Notes:

**File Name:**	3203.del	**Dispersant Name:**	Water
**Record Number:**	3244	**Dispersant RI:**	1.330
**Material RI:**	0.10	**Viscosity (cP):**	0.8872
**Material Absorbtion:**	0.300	**Measurement Date and Time:**	Monday, September 23, 2024...

System

**Temperature (°C):**	25.0	**Duration Used (s):**	70
**Count Rate (kcps):**	303.4	**Measurement Position (mm):**	5.50
**Cell Description:**	Clear disposable zeta cell	**Attenuator:**	11

Results

			Size (d.nm):	% Intensity:	St Dev (d.n...
**Z-Average (d.nm):**	518.6	**Peak 1:**	556.2	70.5	144.8
**PdI:**	0.577	**Peak 2:**	98.75	29.5	25.54
**Intercept:**	0.724	**Peak 3:**	0.000	0.0	0.000

Result quality : **Refer to quality report**

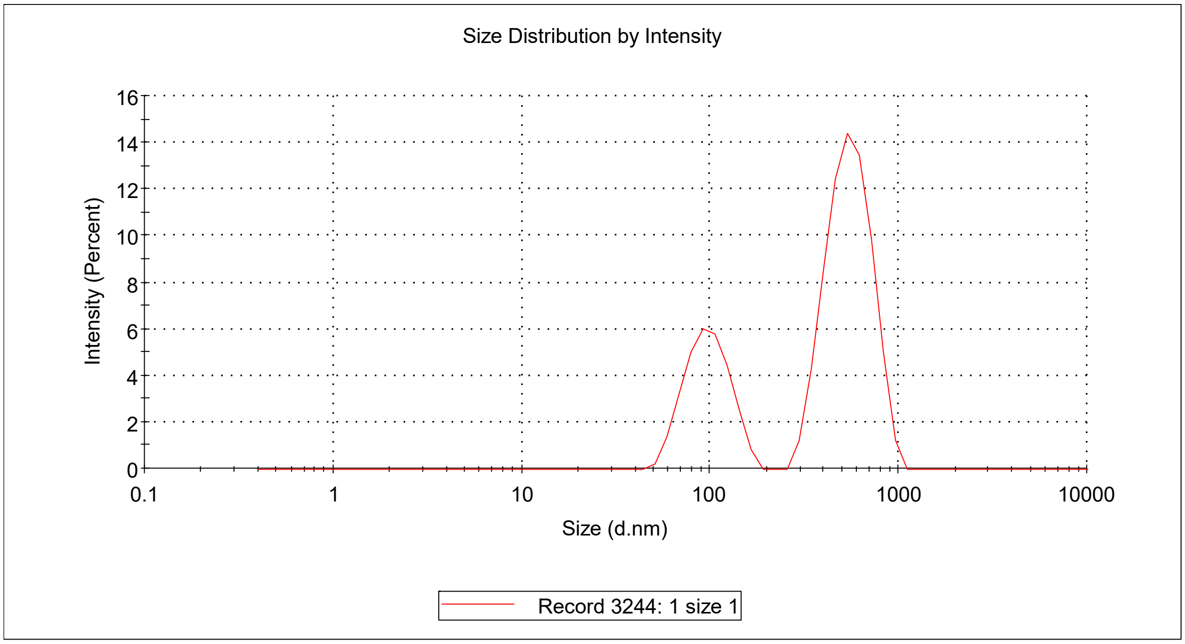

Size Distribution Report by Intensity

v2.2

Supplementary Figure S8

Malvern

Sample Details

Sample Name: 3 size 2

SOP Name: Size Water 0.3

General Notes:

**File Name:**	3203.del	**Dispersant Name:**	Water
**Record Number:**	3263	**Dispersant RI:**	1.330
**Material RI:**	0.10	**Viscosity (cP):**	0.8872
**Material Absorbtion:**	0.300	**Measurement Date and Time:**	Monday, September 23, 2024...

System

**Temperature (°C):**	25.0	**Duration Used (s):**	70
**Count Rate (kcps):**	169.1	**Measurement Position (mm):**	5.50
**Cell Description:**	Clear disposable zeta cell	**Attenuator:**	7

Results

			**Size (d.nm):**	**% Intensity:**	**St Dev (d.n...**
**Z-Average (d.nm):**	256.6	**Peak 1:**	1555	81.7	1339
**PdI:**	1.000	**Peak 2:**	36.26	15.0	12.68
**Intercept:**	0.950	**Peak 3:**	11.58	3.3	2.421

Result quality : **Refer to quality report**

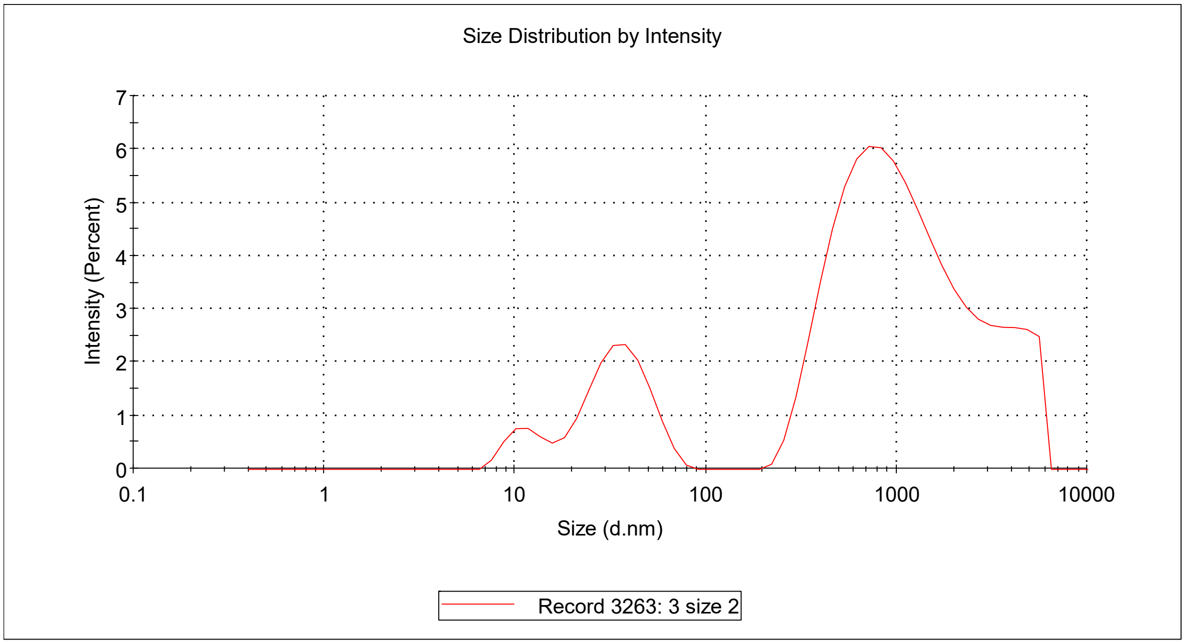

Size Distribution Report by Intensity

v2.2

Supplementary Figure S9

Malvern

Sample Details

**Sample Name:**	Nano Galic size 2
**SOP Name:**	Size Water 0.3
**General Notes:**	

**File Name:**	3203.del	**Dispersant Name:**	Water
**Record Number:**	5326	**Dispersant RI:**	1.330
**Material RI:**	0.10	**Viscosity (cP):**	0.8872
**Material Absorbtion:**	0.300	**Measurement Date and Time:**	Monday, January 20, 2025 1:...

System

**Temperature (°C):**	25.0	**Duration Used (s):**	80
**Count Rate (kcps):**	64.0	**Measurement Position (mm):**	5.50
**Cell Description:**	Clear disposable zeta cell	**Attenuator:**	7

Results

			**Size (d.nm):**	**% Intensity:**	**St Dev (d.n...**
**Z-Average (d.nm):**	128.1	**Peak 1:**	53.12	57.1	31.95
**PdI:**	0.500	**Peak 2:**	370.3	38.6	119.8
**Intercept:**	0.939	**Peak 3:**	5407	4.3	304.4

Result quality : **Refer to quality report**

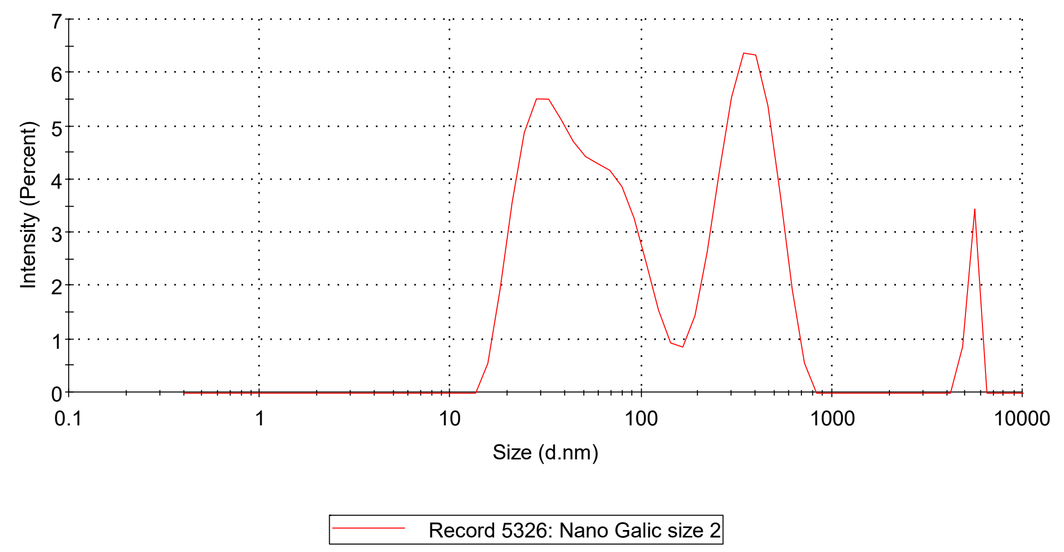

Zeta Potential Report

v2.3

Supplementary Figure S10

Malvern Instruments Ltd - © Copyright 2008

Sample Details

Sample Name: 1 2

SOP Name: Zeta 1.361 water.sop

General Notes:

**File Name:**	3203.del	**Dispersant Name:**	Water
**Record Number:**	3248	**Dispersant RI:**	1.330
**Date and Time:**	Monday, September 23, 2024 11:...	**Viscosity (cP):**	0.8872
		**Dispersant Dielectric Constant:**	78.5

System

**Temperature (°C):**	25.0	**Zeta Runs:**	12
**Count Rate (kcps):**	42.0	**Measurement Position (mm):**	2.00
**Cell Description:**	Clear disposable zeta cell	**Attenuator:**	11

Results

			Mean (mV)	Area (%)	St Dev (mV)
**Zeta Potential (mV):**	-9.76	**Peak 1:**	-9.76	100.0	3.39
**Zeta Deviation (mV):**	3.39	**Peak 2:**	0.00	0.0	0.00
**Conductivity (mS/cm):**	0.143	**Peak 3:**	0.00	0.0	0.00
**Result quality :**	Good				

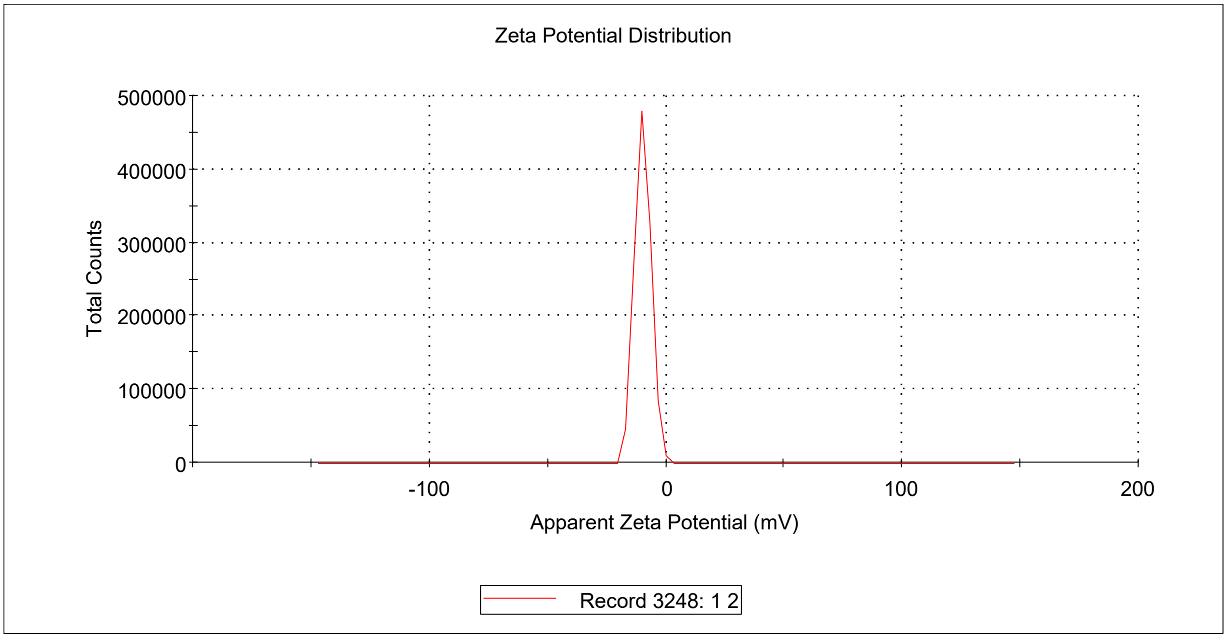

Zeta Potential Report

v2.3

Malvern Instruments Ltd - © Copyright 2008

Supplementary Figure S11

Sample Details

Sample Name: 3 1

SOP Name: Zeta 1.4 w.sop

General Notes:

**File Name:**	3203.del	**Dispersant Name:**	Water
**Record Number:**	3265	**Dispersant RI:**	1.330
**Date and Time:**	Monday, September 23, 2024 12:...	**Viscosity (cP):**	0.8872
		**Dispersant Dielectric Constant:**	78.5

System

**Temperature (°C):**	25.0	**Zeta Runs:**	45
**Count Rate (kcps):**	149.2	**Measurement Position (mm):**	2.00
**Cell Description:**	Clear disposable zeta cell	**Attenuator:**	8

Results

			Mean (mV)	Area (%)	St Dev (mV)
**Zeta Potential (mV):**	-0.716	**Peak 1:**	-0.716	100.0	4.48
**Zeta Deviation (mV):**	4.48	**Peak 2:**	0.00	0.0	0.00
**Conductivity (mS/cm):**	0.435	**Peak 3:**	0.00	0.0	0.00
**Result quality :**	Good				

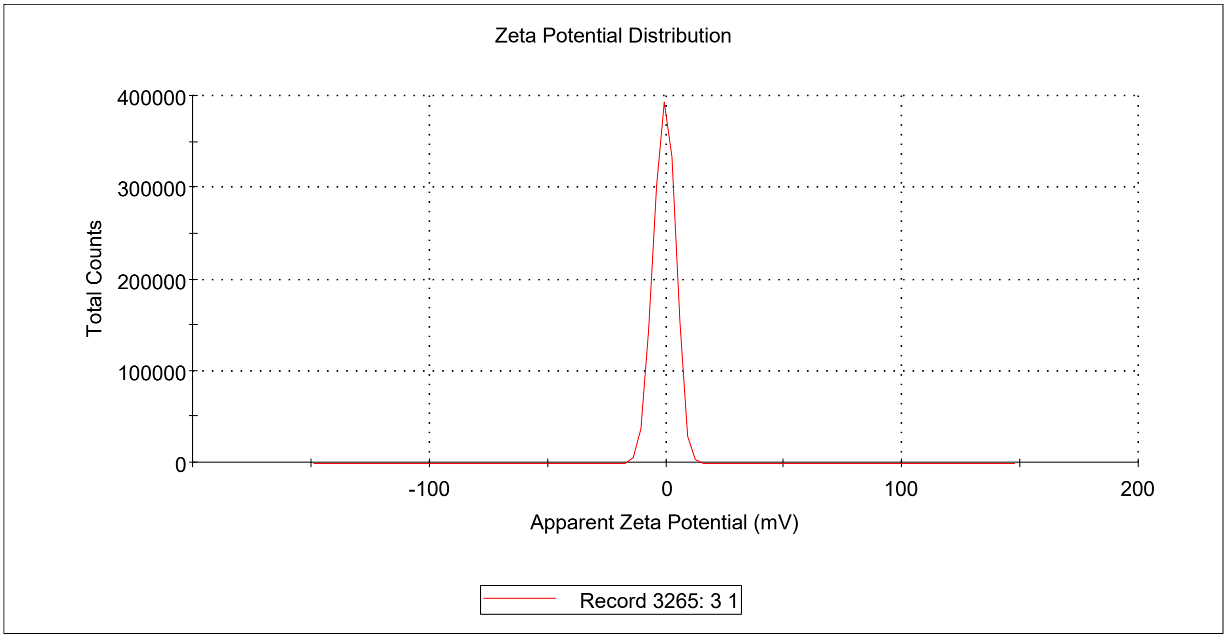

Zeta Potential Report
v2.3

Malvern Instruments Ltd - © Copyright 2008

Supplementary Figure S12

Sample Details

Sample Name: 2 3

SOP Name: R.I 1.49 water.sop

General Notes:

File Name: 3203.del

Record Number: 3255

Date and Time: Monday, September 23, 2024 12:...

Dispersant Name: Water

Dispersant RI: 1.330

Viscosity (cP): 0.8872

Dispersant Dielectric Constant: 78.5

System

Temperature (°C): 25.0

Count Rate (kcps): 643.5

Cell Description: Clear disposable zeta cell

Zeta Runs: 12

Measurement Position (mm): 2.00

Attenuator: 8

Results

Zeta Potential (mV): -14.9

Zeta Deviation (mV): 4.28

Conductivity (mS/cm): 0.122

Result quality : Good

	Mean (mV)	Area (%)	St Dev (mV)
**Peak 1:**	-14.9	100.0	4.28
**Peak 2:**	0.00	0.0	0.00
**Peak 3:**	0.00	0.0	0.00

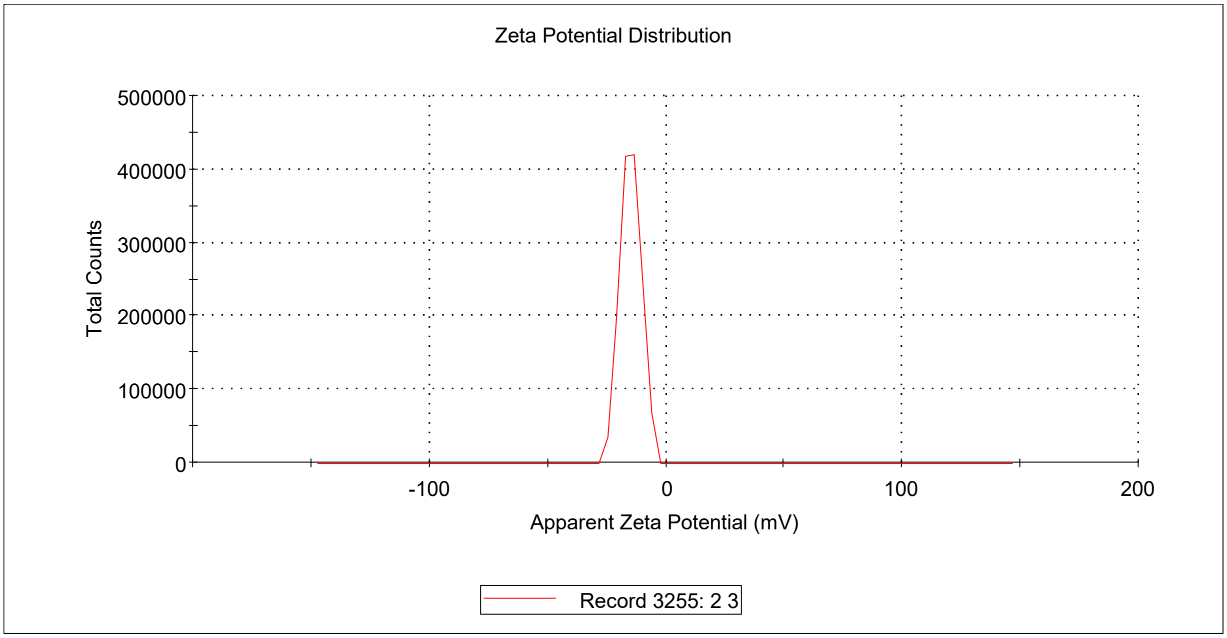

Supplementary Figure S13

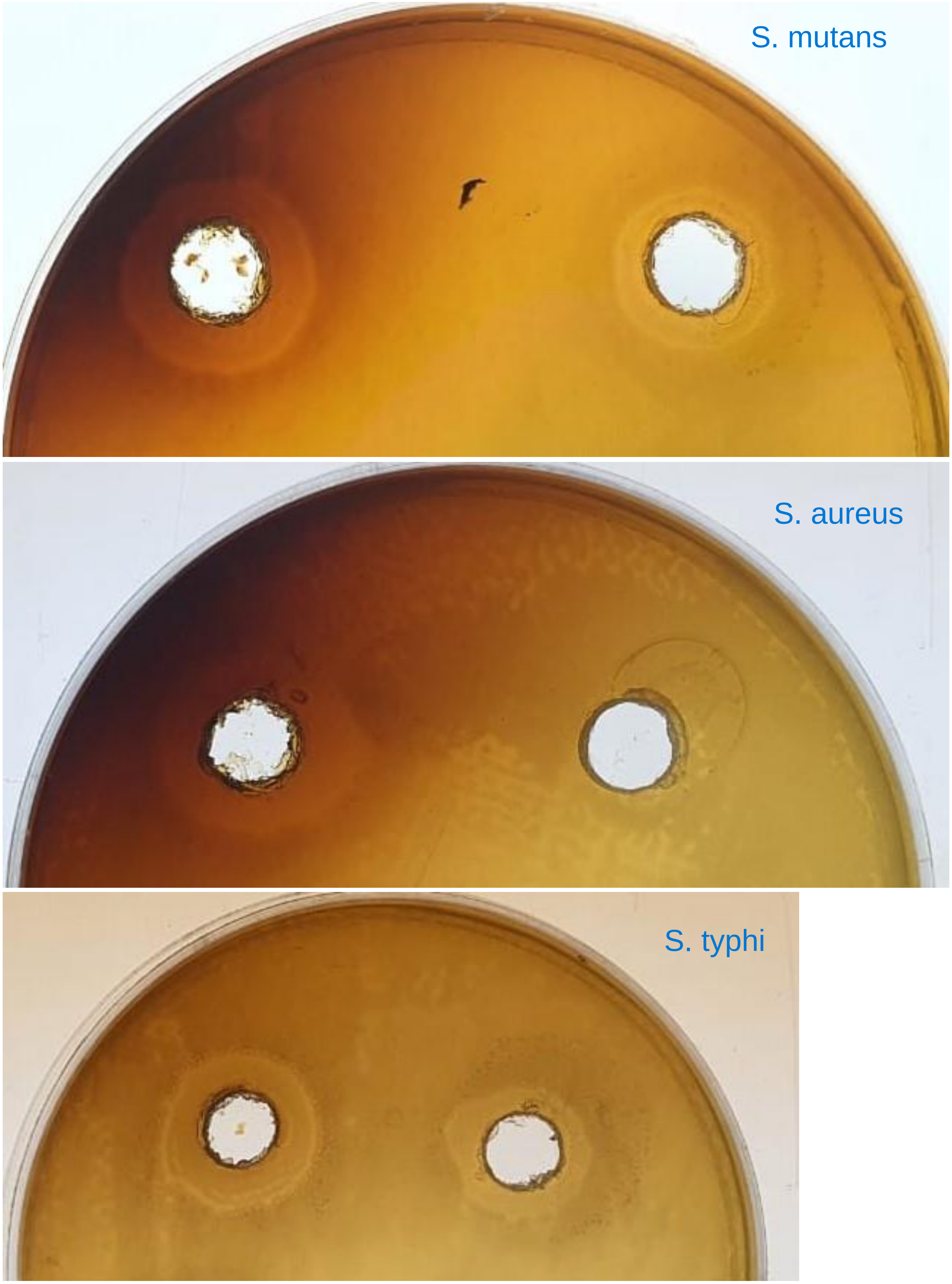

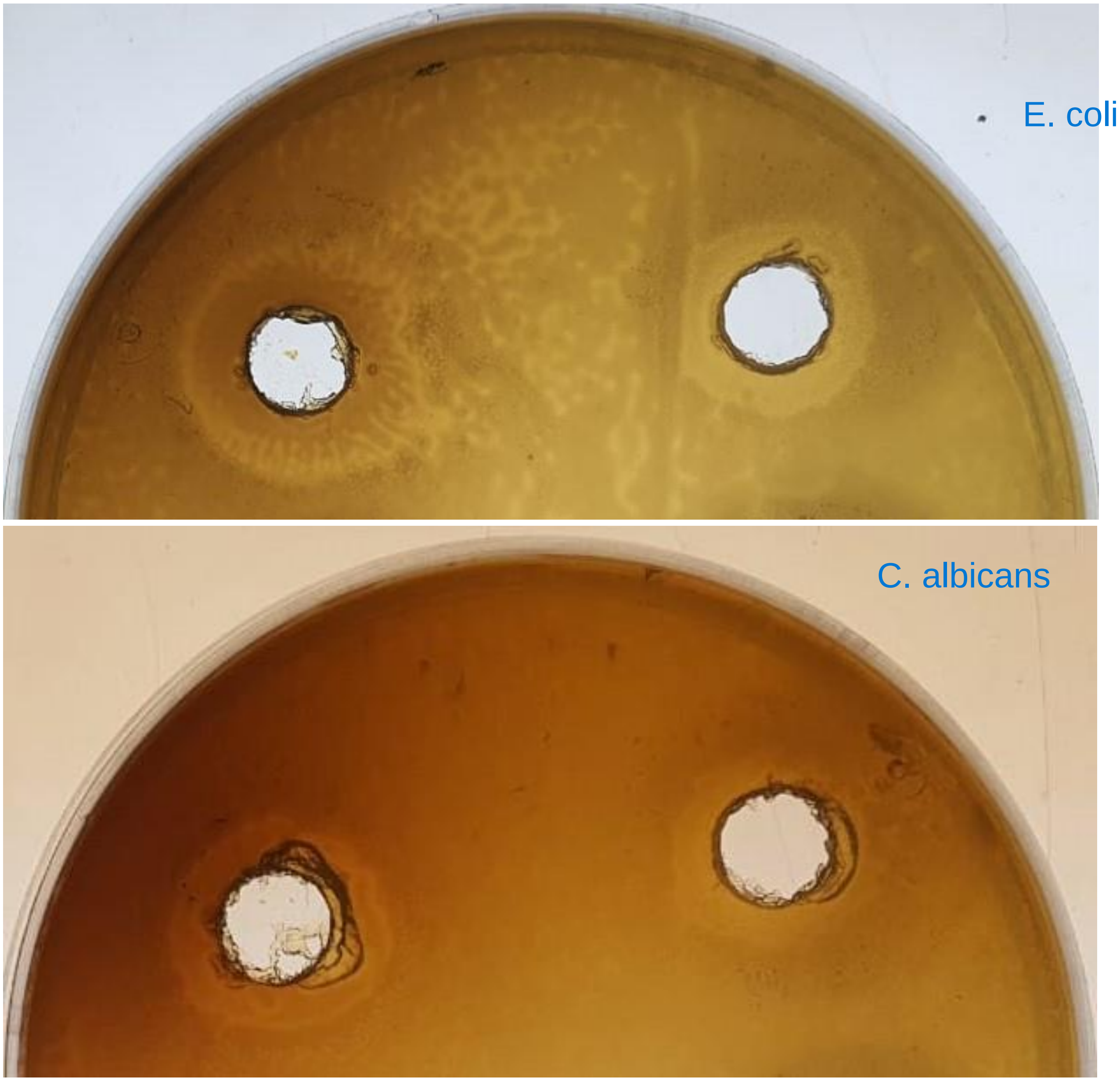
E. coli
C. albicans
