# Supplementary material for: Evaluation of antimicrobial potential of free gallic acid and its polyvinyl-based nano-formulation
Source: Sci Rep. 2025 Oct 1;15:34281. doi: 10.1038/s41598-025-19519-0 (PMC12488905; doi:10.1038/s41598-025-19519-0)
Supplement: Supplementary file 2 — Supplementary Material 2 [file 41598_2025_19519_MOESM2_ESM.docx]

**Supplementary Figures:**

All data obtained from instrumental analyses are provided in the supplementary Figures. TGA results are shown in Supplementary Figures S1–S3 for gallic acid, nano-gallic acid, and PVA, respectively. FTIR spectra are presented in Figures S4–S6 for the same materials. Particle size distribution is shown in Figures S7–S9 for PVA, gallic acid, and nano-gallic acid, respectively. Zeta potential results are provided in Figures S10–S12 for gallic acid, nano-gallic acid, and PVA, respectively. Supplementary Figure S13 includes original images of the antimicrobial inhibition zones against *S. mutans*, *S. aureus*, *S. typhi*, *E. coli*, and *C. albicans*.
